# Preventive efficacy of six monthly doses of NexGard® PLUS or Simparica Trio® against a macrocyclic lactone-resistant isolate (JYD-34) of *Dirofilaria immitis* and of a single dose of NexGard PLUS against a susceptible isolate

**DOI:** 10.1186/s13071-024-06603-z

**Published:** 2024-12-18

**Authors:** Joseph Prullage, Justin Frost, Utami DiCosty, Elizabeth Martin, Pascal Dumont, Stephen Yoon, Ricarda Süssenberger

**Affiliations:** 1 Boehringer Ingelheim Animal Health, Missouri Research Center, 6498 Jade Rd., Fulton, MO 65251 USA; 2 Boehringer Ingelheim Animal Health, Georgia Research Center, 1730 Olympic Dr., Athens, GA 30601 USA; 3 TRS Labs, Inc., 215 Paradise Boulevard, Athens, GA 30607 USA; 4 Boehringer Ingelheim Vetmedica GmbH, Binger Str. 173, 55216 Ingelheim am Rhein, Germany

**Keywords:** Canine, Moxidectin, *Dirofilaria immitis*, Prevention, Macrocyclic lactone-resistant JYD-34 isolate, Susceptible SC-20 isolate

## Abstract

**Background:**

Two studies were conducted assessing the efficacy of NexGard® PLUS (NP) in preventing heartworm disease. Study 1 evaluated the efficacy of six monthly doses of NP or Simparica Trio® (ST) against a macrocyclic lactone-resistant isolate of heartworm, *Dirofilaria immitis*, and study 2 evaluated the efficacy of a single dose of NP against a susceptible isolate.

**Methods:**

In two studies, dogs that were negative for heartworms by antigen test and modified Knott’s test were used. In study 1, dogs were randomly allocated into three treatment groups (*n* = 6/group): negative control, NP per label instructions, and ST per label instructions. Dogs were inoculated with 50 third-stage *D. immitis* larvae (JYD-34 isolate) on day −30. NP and ST were administered orally on days 0, 30, 60, 90, 120, and 150. A necropsy was performed on day 180 for adult heartworm recovery. In study 2, dogs were randomly allocated into two treatment groups (*n* = 10/group): negative control and NP. Dogs were inoculated with 50 third-stage larvae (SC-20 isolate) on day −30. NP was administered orally once on day 0 to target the minimum moxidectin label dose. A necropsy was performed on day 120 for adult heartworm recovery.

**Results:**

For study 1, all control dogs had adult heartworms at necropsy (geometric mean, 39.7; range, 28–48). Two of the NP-treated dogs had one live worm, and one of the ST-treated dogs had one live worm. Both treated groups were significantly different from the control group with an efficacy of 99.5% for NP and 99.8% for ST (*P* < 0.0001). There was no significant difference (*P* = 0.8797) between the groups treated with NP and ST. For study 2, all control dogs had adult heartworms (geometric mean, 34.5; range 26–43). None of the dogs treated with NP had live adult worms (efficacy of 100%, *P* < 0.0001).

**Conclusions:**

The results of study 1 demonstrated that NexGard® PLUS and Simparica Trio® administered at the label dose provided comparable efficacy against a macrocyclic lactone-resistant isolate of *D. immitis.* The results of study 2 demonstrated that NexGard® PLUS administered once near the minimum label dose was 100% effective against a susceptible isolate.

**Graphical Abstract:**

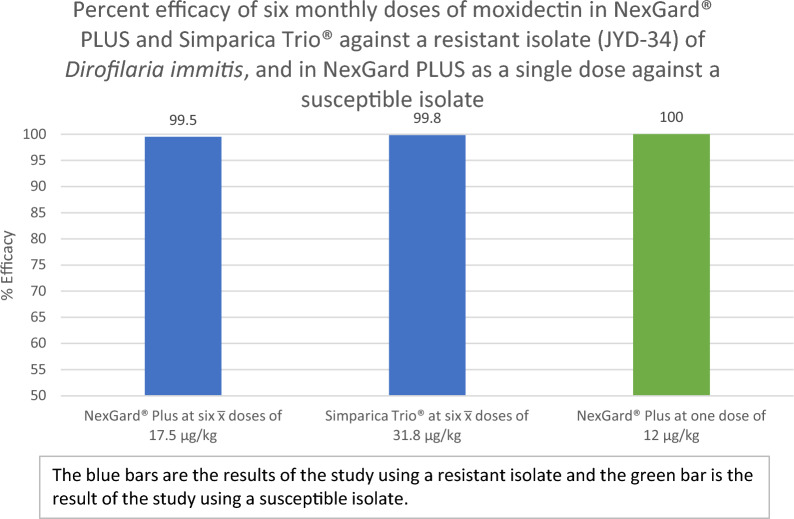

## Background

Heartworm disease, caused by the filarial nematode *Dirofilaria immitis*, is a chronic and potentially fatal cardiopulmonary condition found in dogs worldwide. Mosquitoes serve as the vector for *D. immitis*, with 32 species in the USA found to be naturally infected or capable of supporting development to the third larval stage (L3) [1, 2]. The prevalence of canine heartworm disease is increasing, and its geographic range is expanding, within parts of the USA, including areas where the risk historically was believed to be relatively low [4]. This trend is attributed to several factors, including inadequate use of heartworm preventives, changes in climatic conditions, proliferation and spread of mosquito vectors, increased movement of heartworm-infected dogs, and presence and expansion of wildlife reservoir populations [3–5].

Given the severity and widespread distribution of heartworm disease, its prevention remains a primary focus in companion animal health. Emphasizing prevention over treatment is crucial due to the significant cardiopulmonary damage caused by both live and dead nematodes, as well as the challenges associated with adulticidal treatment, such as prolonged drug therapy, exercise restrictions, lung pathology, and potential complications. At this time, macrocyclic lactones (MLs) remain the only class of commercially available compounds used in drug products for the prevention of heartworm disease, and the MLs remain highly effective when used appropriately.

In recent years, the recognition of ML resistance in the *D. immitis* population has led to renewed interest and research related to heartworm disease and its prevention. The resistance of several isolates of *D. immitis* to MLs has been confirmed in laboratory settings [6–8]. While these isolates are resistant to all drugs within the ML class, moxidectin has repeatedly demonstrated beneficial properties that have shown higher efficacy in laboratory studies among the commercially available MLs [9–12].

In 1997, the original oral moxidectin heartworm preventive product was approved with a minimum oral moxidectin dose of 3 μg/kg. More recently, studies conducted against the ML-resistant JYD-34 heartworm isolate using a single oral dose of moxidectin resulted in an efficacy of 19%, and a single oral dose as high as 100 μg/kg was only 91.1% effective [10, 13].

To further explore the impact of moxidectin dosing, studies were conducted to assess the efficacy of moxidectin dose used in NexGard® PLUS (with a minimum recommended moxidectin dose of 12 μg/kg) in preventing heartworm disease in two very different scenarios. Study 1 compared the preventive heartworm efficacy of six monthly doses of NexGard® PLUS chewable tablets or Simparica Trio® chewable tablets, both at their approved labeled dosages against the confirmed ML-resistant isolate, JYD-34. Study 2 evaluated the efficacy of a single dose of NexGard® PLUS chewable tablets at its minimum labeled dose (i.e., 12 µg/kg) against a known ML-susceptible heartworm isolate.

## Methods

In both studies, dogs were acclimated to the study conditions for 7 days and managed similarly and with due regard for their well-being. The study design was reviewed and approved by the Sponsor’s and local institutional animal care and use committees and met USDA-APHIS (United States Department of Agriculture-Animal and Plant Health Inspection Service) animal welfare requirements. A physical examination was conducted during acclimation, and dogs were observed for general health at least once daily throughout the study.

### Animal model and treatment

#### Comparison of NexGard® PLUS to Simparica Trio® (study 1)

Eighteen healthy purpose-bred Beagle dogs, 8 male and 10 female, approximately 9 months old, weighing 7.3–11.1 kg on day −2 and never treated with an ML or tetracycline antibiotic were included. The study used a randomized block design with randomization restricted so that each group had at least two males and two females. Dogs were randomly assigned to cages and rooms. A randomization order was also followed for euthanasia, necropsy, and heartworm counts. Dogs were individually housed 1 day prior to and at least 7 days following each treatment. Dogs were housed in pairs within group and sex as temperament allowed outside of these times to prevent cross-contamination between treatment groups by moxidectin eliminated in the feces. The PLAN procedure of SAS version 9.4 was used for all randomizations.

There were three treatment groups: a sham-dosed control, NexGard® PLUS (Boehringer Ingelheim), and Simparica Trio® (Zoetis). The dogs in the control group were sham dosed. Dogs in the NexGard® PLUS- and Simparica Trio®-treated groups were treated with the appropriate chewable tablet according to the weight bands on the product label, providing for NexGard® PLUS 11.69–23.08 µg/kg moxidectin, 2.4–5.2 mg/kg afoxolaner, 4.9–10.3 mg/kg pyrantel pamoate, and for Simparica Trio® 24–48 µg/kg moxidectin, 1.2–2.4 mg/kg sarolaner, 5.0–10.0 mg/kg pyrantel pamoate. Dogs were treated every 30 days starting on day 0 for six treatments (actual dose ranges are summarized in Table 1).

**Table 1:** Treatment details

Treatment group	Study 1		Study 2	
	Body weight range day 0 (kg)	Moxidectin mean and (dose range)^a^ (µg/kg)	Body weight range day 0 (kg)	Moxidectin mean and (dose range) (µg/kg)
Sham-dosed control	7.6–10.4	Not applicable	7.44–9.50	Not applicable
NexGard® PLUS	7.6–11.3	17.52 (11.69–23.08)	6.98–9.98	12.64 to 17.40
Simparica Trio®	7.7–9.9	31.76 (24.24–47.52)	Not applicable	Not applicable

^a^The mean and dose range reported for moxidectin is for all six treatments of each dog in each treatment group

#### Single administration study (study 2)

Twenty healthy purpose-bred Beagle dogs, 10 male and 10 female, 7.3–8.0 months old, weighing 6.8–10.0 kg and not treated with an ML or tetracycline antibiotic were used. Dogs were ranked by decreasing body weight within sex, and ten blocks of two dogs each were formed. Within blocks, dogs were randomly assigned to a treatment group with ten dogs assigned to the untreated control and ten dogs assigned to the NexGard® PLUS (Boehringer Ingelheim)-treated group. Dogs were randomly assigned to pens, and to an order for euthanasia, necropsy, and heartworm counts. Dogs were housed individually to prevent cross-contamination by moxidectin eliminated in the feces. The PLAN procedure of SAS version 9.4 was used for all randomizations.

A combination of chews of NexGard® PLUS was administered once on day 0 to provide a dose of moxidectin as close as possible to the minimum recommended dose of 12 µg/kg (actual dose range is summarized in Table 1).

In both studies, all personnel involved with evaluation of effectiveness (heartworm counts) and recording of health observations were masked to treatment assignments. Dogs were infected with 50 *D. immitis* third-stage larvae (L3) by subcutaneous injection on day −30. For the study comparing NexGard® PLUS with Simparica Trio®, the L3 used for the experimental inoculations were from the JYD-34 isolate maintained in colony by TRS Labs, Inc. (Athens, GA, USA). The JYD-34 isolate originated in Illinois in July 2010 and was in its third passage. For the study using a single administration of NexGard® PLUS, the L3 used for the experimental inoculations were from the SC-20 laboratory isolate maintained by TRS Labs, Inc. In both studies, heartworm antigen tests were conducted on day 90 to confirm that dogs were not infected with *D. immitis* prior to the start of the study.

Dogs were euthanized on day 180 in study 1 comparing NexGard® PLUS with Simparica Trio® and on day 120 in study 2 assessing a single administration of NexGard® PLUS. In both studies, an incision was made at the site where the L3 were injected and the area searched for heartworms. The abdominal cavity was opened and also searched for heartworms. The thoracic cavity was opened, the anterior and posterior vena cava were clamped, the heart and lungs were removed together, and the thoracic cavity was searched for heartworms. The heart and veins in the lungs were dissected and searched for heartworms. Heartworm fragments, if any, were counted as follows: worm fragments containing a head and worm fragments containing a tail were counted separately. The greater of the two counts (number of fragments containing a head or number of fragments containing a tail) were included in the total worm count for effectiveness calculations. Total counts of live and dead heartworms were recorded for all sites for each dog.

### Statistical methods

#### Study 1

Counts of *D. immitis* were transformed to the natural logarithm of (count + 1) for the comparison of the NexGard® PLUS-treated group against the sham-dosed control group, the Simparica Trio®-treated group against the sham-dosed control group and the comparison of the NexGard® PLUS group against the Simparica Trio®-treated group. The MIXED procedure of SAS version 9.4 was used for the analysis of the log-counts with the treatment group, sex, and sex-by-treatment group interaction included as fixed effects. Pairwise *t*-tests were used to test for significance between treatment groups. Tukey’s adjustment method for multiple pairwise comparisons was used to control the overall significance level for all pairwise comparisons. Efficacy was determined for adult *D. immitis* by calculating the percent efficacy as 100[(*C* − *T*)/*C*], where *C* is the back-transformed least squares mean of the untreated control group obtained from the model and *T* is the back-transformed least squares means of the treated groups.

#### Study 2

Counts of *D. immitis* were transformed to the natural logarithm of (count + 1) for the comparison of the NexGard® PLUS-treated group against the untreated control group. The MIXED procedure of SAS version 9.4 was used for the analysis of the log-counts with the treatment group, sex, and sex-by-treatment group interaction included as fixed effects, and block as a random effect. The *F*-test was used to test for significance between treatment groups. Efficacy was determined for adult *D. immitis* by calculating the percent efficacy as 100[(*C* − *T*)/*C*], where *C* is the back-transformed least squares mean of the untreated control group obtained from the model and *T* is the back-transformed least squares mean of the treated group.

## Results

### Study 1

Heartworm counts and analyses are presented in Table 2. All dogs in the control group were infected with heartworms, with counts ranging from 28 to 48 heartworms, with a geometric mean of 39.7 heartworms. There was one heartworm each recovered from two dogs in the NexGard® PLUS-treated group and one heartworm recovered from one dog in the Simparica Trio®-treated group. Percent efficacy was 99.5% for NexGard® PLUS and 99.8% for Simparica Trio®. The heartworm counts for the NexGard® PLUS- and the Simparica Trio®-treated groups were significantly different from the control group (*P* < 0.0001). Comparison of the NexGard® PLUS treated group with the Simparica Trio® treated group did not show a significant difference between these groups (*P* = 0.8797).

**Table 2:** Counts of live *D. immitis* and statistical analysis of the results

Study	Treatment group	Number of dogs	Number of dogs with heartworms	Range of heartworm counts	Geometric mean of heartworm counts	Percent efficacy^a^	*P* value^b^
1	Sham-dosed control	6	6	28–48	39.7	Not applicable	
	NexGard® PLUS	6	2	1	0.2	99.5	< 0.0001
	Simparica Trio®	6	1	1	0.1	99.8	< 0.0001
2	Sham-dosed control	10	10	26–43	34.5	Not applicable	
	NexGard® PLUS	10	0	Not applicable	0.0	100	< 0.0001

^a^Percent efficacy = 100[(*C* − *T*)/*C*], where *C* and *T* are the geometric means calculated from the back-transformed least squares means of each treated group and untreated control group, respectively

^b^*P* values: for study 1, two-sided probability value from pair-wise *t*-tests using Tukey’s adjustment method on log-counts; for study 2: two-sided probability value from analysis of variance on log-counts of treated group and untreated control group

On day 150 of the study, one dog in the NexGard® PLUS-treated group vomited after 2 h post-treatment and at least part of the chew was found in the vomit. This was not readministered as it was later than 2 h after treatment as defined in the protocol of the study. Other health abnormalities noted during the study were minor, and the incidence was similar between treatment groups. These included intermittent vomiting and diarrhea, abrasions/wounds, dermatitis, alopecia, and lameness.

### Study 2

Heartworm counts and analyses are presented in Table 2. Dogs in the control group had a geometric mean of 34.5 live adult *D. immitis* (26–43 worms with 10 of 10 dogs infected). There were no heartworms recovered from any of the dogs in the NexGard® PLUS-treated group, therefore demonstrating 100% efficacy of the 12 µg/kg dose of moxidectin in NexGard® PLUS for the prevention of heartworm disease. The heartworm counts for the NexGard® PLUS-treated group were significantly different from the control group (*P* < 0.0001).

Minor health abnormalities, including interdigital cysts and dermatological abnormalities, were reported in both groups and were not related to treatment. No adverse events related to treatment were observed in any of the NexGard® PLUS-treated dogs.

## Discussion

Results from study 2 demonstrated that a single dose of NexGard® PLUS at or near the minimum dose of 12 µg/kg was fully effective in the prevention of heartworm disease against a susceptible isolate. Previous studies with orally administered moxidectin at 3 µg/kg have also shown 100% efficacy against ML-susceptible *D. immitis* isolates in the prevention of heartworm disease [10, 11, 14]. Moxidectin is the most potent of the MLs, and it exhibits the highest lipophilicity, allowing for a longer half-life and increased tissue distribution. Physiochemical and pharmacokinetic properties are important in modulating the rate of drug exchange between the bloodstream and tissues where the larval heartworms are developing and likely explain moxidectin’s improved performance against known ML resistant *D. immitis* isolates compared with other MLs [15].

As resistance in *D. immitis* to the ML class of anthelmintics will likely continue to develop and spread, it has become important to also determine the efficacy of products against resistant isolates. To this end, the study of NexGard® PLUS and Simparica Trio® compared two target doses of moxidectin: 12 µg/kg for NexGard® PLUS and 24 µg/kg for Simparica Trio® when given for six monthly treatments. Despite the difference in moxidectin dose, NexGard® PLUS and Simparica Trio® provided comparable levels of control of the JYD-34 isolate of *D. immitis* (99.5% for NexGard® PLUS and 99.8% for Simparica Trio®) after six monthly administrations. Counts of heartworms were not significantly different between the NexGard® PLUS- and Simparica Trio®-treated groups (*P* = 0.8797). These results are similar to previous studies using multiple treatments of moxidectin against known ML-resistant isolates of *D. immitis* [13, 16]. However, previous studies tested doses that were below 12 µg/kg or at or above 24 µg/kg. Results from study 1 demonstrated that six monthly doses of 11.69–23.08 µg/kg (mean 17.5 µg/kg) is as effective as six monthly doses of 24.24–47.52 (mean 31.8 µg/kg) of moxidectin.

These results raise the question of whether the dose of moxidectin or the number of doses is the more critical factor in preventing heartworm disease caused by ML-resistant *D. immitis*. Savadelis et al. [16] proposed that management of ML resistance was best achieved by optimized formulations with increased doses. The results of this study indicate that regular monthly administration of the preventive is likely as important as raising the dose of moxidectin from the original 3 µg/kg dose for the prevention of heartworm disease caused by resistant *D. immitis*.

## Conclusions

The results of study 1 comparing the doses of moxidectin in NexGard® PLUS and Simparica Trio® demonstrated that NexGard® PLUS and Simparica Trio® administered at their respective label doses for six monthly treatments provided comparable efficacy against an isolate known to be resistant to MLs. Study 1 also indicates that repeated doses of moxidectin may be as important as elevated doses of moxidectin for the prevention of heartworm disease caused by ML-resistant *D. immitis*. Results from study 1 using a single administration of NexGard® PLUS demonstrated that the 12 µg/kg dose was 100% effective against ML-susceptible *D. immitis*.

## Data Availability

Data are provided within the manuscript.

## Abbreviations

JYD-34: JYD-34 isolate of *Dirofilaria immitis*
ML: Macrocyclic lactone
NP: NexGard® PLUS
SC-20: SC-20 isolate of *Dirofilaria immitis*
SP: Simparica Trio®

## References

[1] Scoles G. Vectors of canine heartworm in the United States: a review of the literature including new data from Indiana, Florida, and Louisiana, recent advances in heartworm disease: symposium’98. In: Proceedings of the American Heartworm Society*.* Tampa: American Heartworm Society; 1998. p. 21–36.

[2] Ledesma N, Harrington L. Mosquito vectors of dog heartworm in the United States: vector status and factors influencing transmission efficiency. Top Companion Anim Med. 2011;26:178–85. 10.1053/j.tcam.2011.09.005. 22152605 10.1053/j.tcam.2011.09.005

[3] Bowman DD, Liu Y, McMahan CS, Nordone SK, Yabsley MJ, Lund RB. Forecasting United States heartworm *Dirofilaria immitis* prevalence in dogs. Parasit Vectors. 2016;9:540. 10.1186/s13071-016-1804-y. 27724981 10.1186/s13071-016-1804-y PMC5057216

[4] Self SW, Pulaski CN, McMahan CS, Brown DA, Yabsley MJ, Gettings JR. Regional and local temporal trends in the prevalence of canine heartworm infection in the contiguous United States: 2012–2018. Parasit Vectors. 2019;12:380. 10.1186/s13071-019-3633-2. 31362754 10.1186/s13071-019-3633-2 PMC6668072

[5] Noack S, Harrington J, Carithers DS, Kaminsky R, Selzer PM. Heartworm disease—overview, intervention, and industry perspective. Int J Parasitol Drugs Drug Resist. 2021;16:65–89. 10.1016/j.ijpddr.2021.03.004. 34030109 10.1016/j.ijpddr.2021.03.004 PMC8163879

[6] Bourguinat C, Keller K, Prichard RK, Geary TG. Genetic polymorphism in *Dirofilaria immitis*. Vet Parasitol. 2011;176:368–73. 10.1016/j.vetpar.2011.01.023. 21310534 10.1016/j.vetpar.2011.01.023

[7] Pulaski CN, Malone JB, Bourguinat C, Prichard R, Geary T, Ward D, et al. Establishment of macrocyclic lactone resistant *Dirofilaria immitis* isolates in experimentally infected laboratory dogs. Parasit Vectors. 2014;7:494. 10.1186/s13071-014-0494-6. 25376278 10.1186/s13071-014-0494-6 PMC4228187

[8] Blagburn BL, Arther RG, Dillon AR, Butler JM, Bowles JV, von Simson C, et al. Efficacy of four commercially available heartworm preventive products against the JYD-34 laboratory strain of *Dirofilaria immitis*. Parasit Vectors. 2016;9:191. 10.1186/s13071-016-1476-7. 27044379 10.1186/s13071-016-1476-7 PMC4820942

[9] Bowman DD, McTier TL, Adams EL, Magabir SP, Login JA, Bidgood T, et al. Evaluation of the efficacy of ProHeart® 6 (moxidectin) against a resistant isolate of *Dirofilaria immitis* (JYD-34) in dogs. Parasit Vectors. 2017;10:502. 10.1186/s13071-017-2431-y. 29143654 10.1186/s13071-017-2431-y PMC5688425

[10] McTier TL, Six RH, Pullins A, Chapin S, McCall JW, Rugg D, et al. Efficacy of oral moxidectin against susceptible and resistant isolates of *Dirofilaria immitis* in dogs. Parasit Vectors. 2017;10:482. 10.1186/s13071-017-2429-5. 29143634 10.1186/s13071-017-2429-5 PMC5688394

[11] McTier TL, Six RH, Pullins A, Chapin S, Kryda K, Mahabir SP, et al. Preventive efficacy of oral moxidectin at various doses and dosage regimens against macrocyclic lactone-resistant heartworm (*Dirofilaria immitis*) strains in dogs. Parasit Vectors. 2019;12:444. 10.1186/s13071-019-3685-3. 31506088 10.1186/s13071-019-3685-3 PMC6737633

[12] Kryda K, Holzmer SJ, Everett WR, McCall JW, Mahabir SP, McTier TL, et al. Preventive efficacy of four or six monthly oral doses of 24 μg/kg moxidectin compared to six monthly doses of Heartgard® PLUS or Interceptor® PLUS against macrocyclic lactone-resistant heartworm (*Dirofilaria immitis*) strains in dogs. Parasit Vectors. 2020;13:339. 10.1186/s13071-020-04178-z. 32660542 10.1186/s13071-020-04178-z PMC7359479

[13] Martin EM, Mitchell EB, Yoon S, McCall JW, Fankhauser B, Mansour A, et al. Efficacy of moxidectin, using various dose regimens, against JYD-34, a macrocyclic lactone resistant isolate of *Dirofilaria immitis*. Parasit Vectors. 2024;17:176. 10.1186/s13071-024-06149-0. 38575969 10.1186/s13071-024-06149-0 PMC10996163

[14] McTier TL, McCall JW, Dzimianski MT, Aguilar R, Wood I. Prevention of experimental infection in dogs with single, oral doses of moxidectin. In: Proceedings of the Heartworm Symposium ʼ92. Batavia: American Heartworm Society; 1992. p. 165–8.

[15] Prichard RK, Geary TG. Perspectives on the utility of moxidectin for the control of parasitic nematodes in the face of developing anthelmintic resistance. Int J Parasitol Drugs Drug Resist. 2019;10:69–83. 10.1016/j.ijpddr.2019.06.002. 31229910 10.1016/j.ijpddr.2019.06.002 PMC6593148

[16] Savadelis MD, McTier TL, Kryda K, Maeder SJ, Woods DJ. Moxidectin: heartworm disease prevention in dogs in the face of emerging macrocyclic lactone resistance. Parasit Vectors. 2022;15:82. 10.1186/s13071-021-05104-7. 35277180 10.1186/s13071-021-05104-7 PMC8915515

